# Supplementary figures and images for: A preliminary case study of the effect of shoe-wearing on the biomechanics of a horse’s foot
Source: PeerJ. 2016 Jul 14;4:e2164. doi: 10.7717/peerj.2164 (PMC4950542; doi:10.7717/peerj.2164)

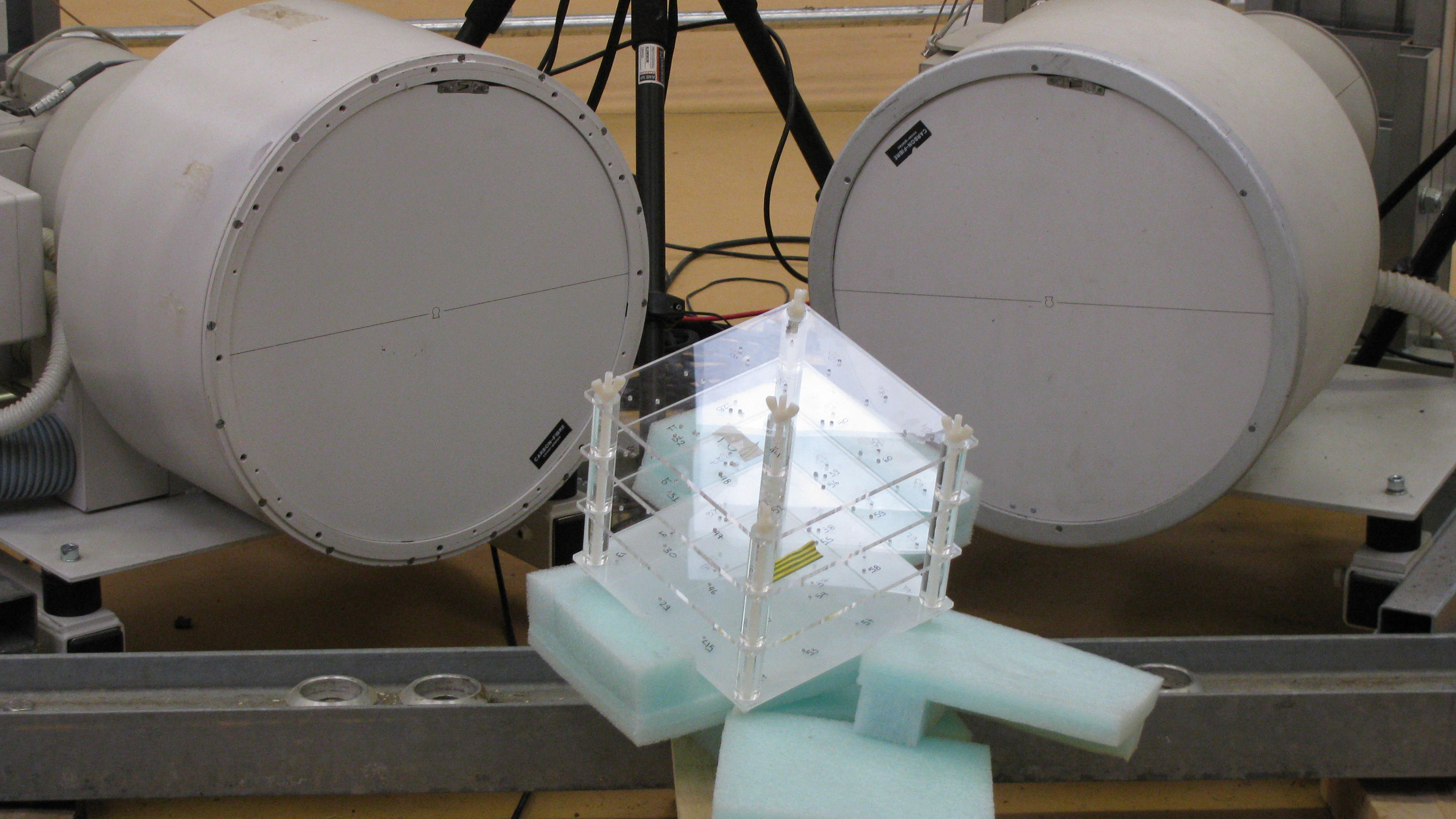

Supplement: Figure S1 [file peerj-04-2164-s001.jpg]

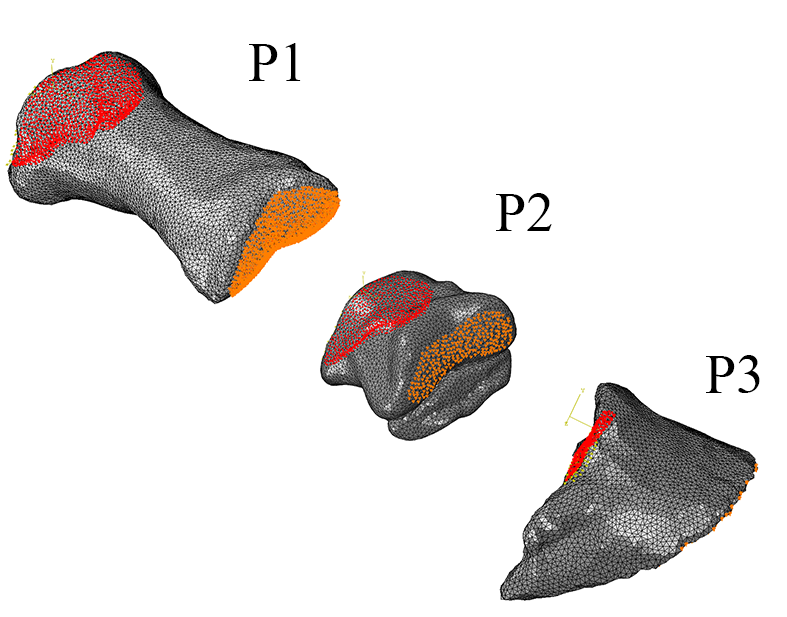

Supplement: Figure S2 [file peerj-04-2164-s002.png]

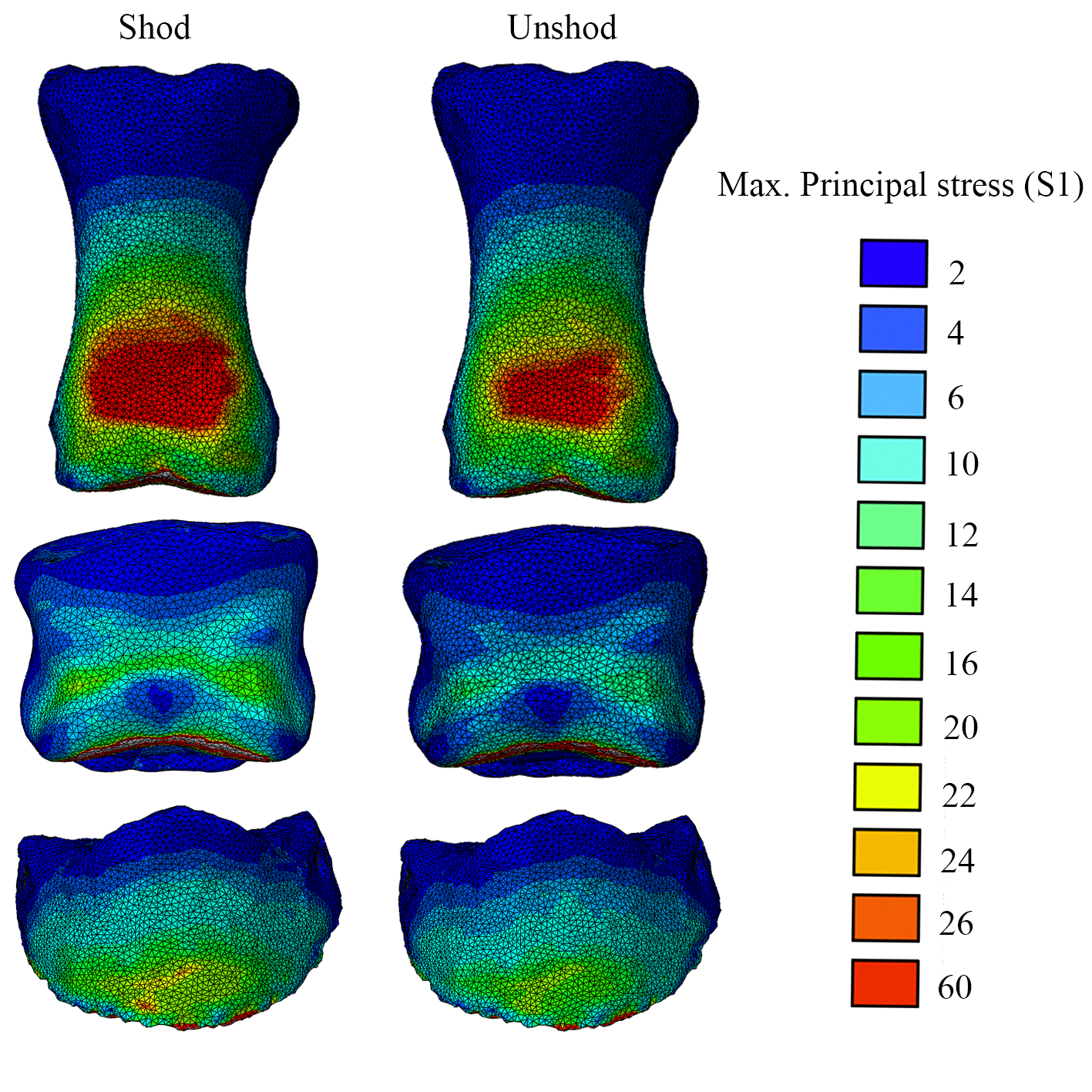

Supplement: Figure S3 — Bones shown from top to bottom are the P1, P2 and P3. Warm (red) and cold (blue) colours show higher and lower maximum principal stresses respectively. [file peerj-04-2164-s003.png]

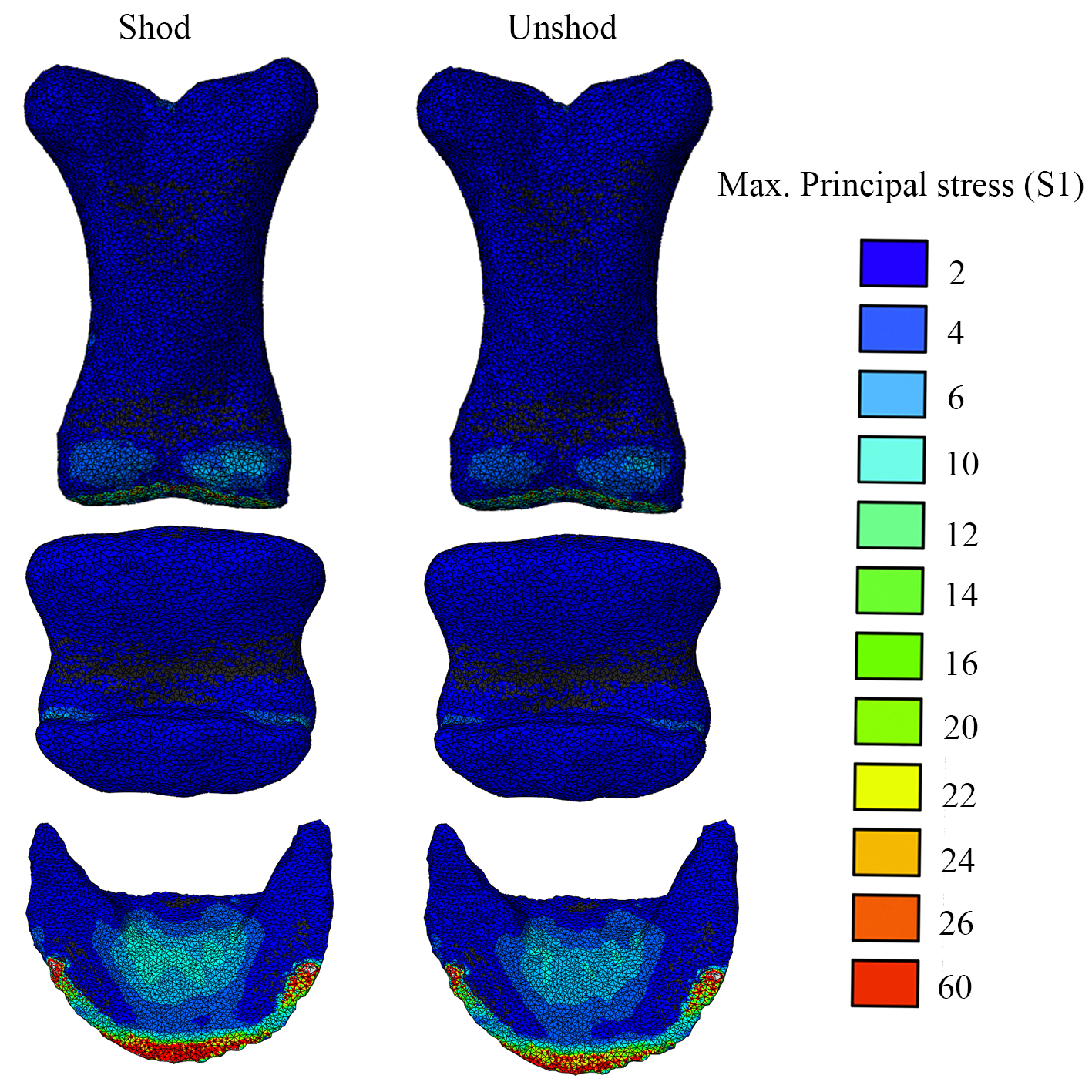

Supplement: Figure S4 — Bones shown from top to bottom are the P1, P2 and P3. Warm (red) and cold (blue) colours show higher and lower maximum principal stresses respectively. [file peerj-04-2164-s004.png]

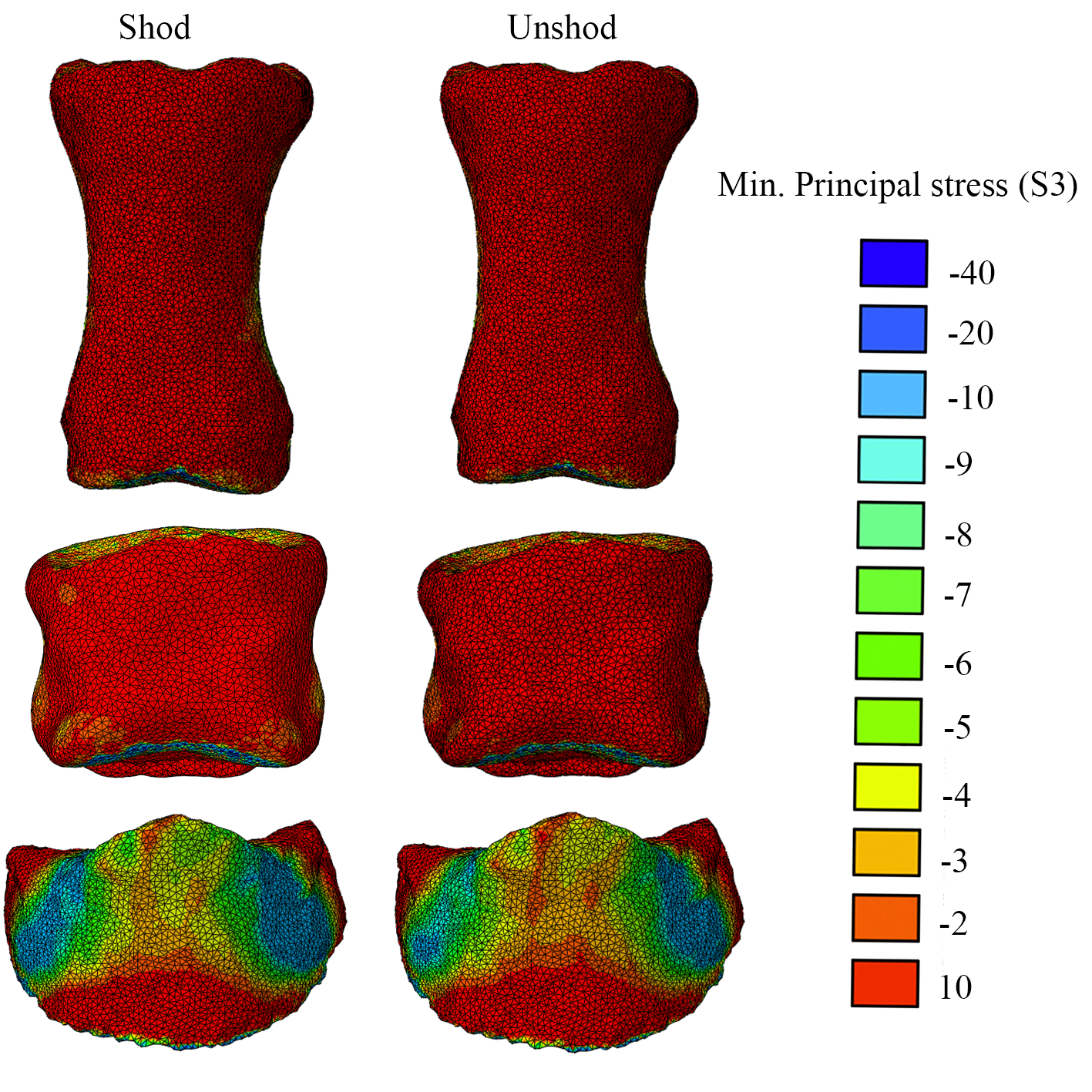

Supplement: Figure S5 — Bones shown from top to bottom are the P1, P2 and P3. Warm (red) and cold (blue) colours show higher and lower minimum principal stresses respectively. [file peerj-04-2164-s005.png]

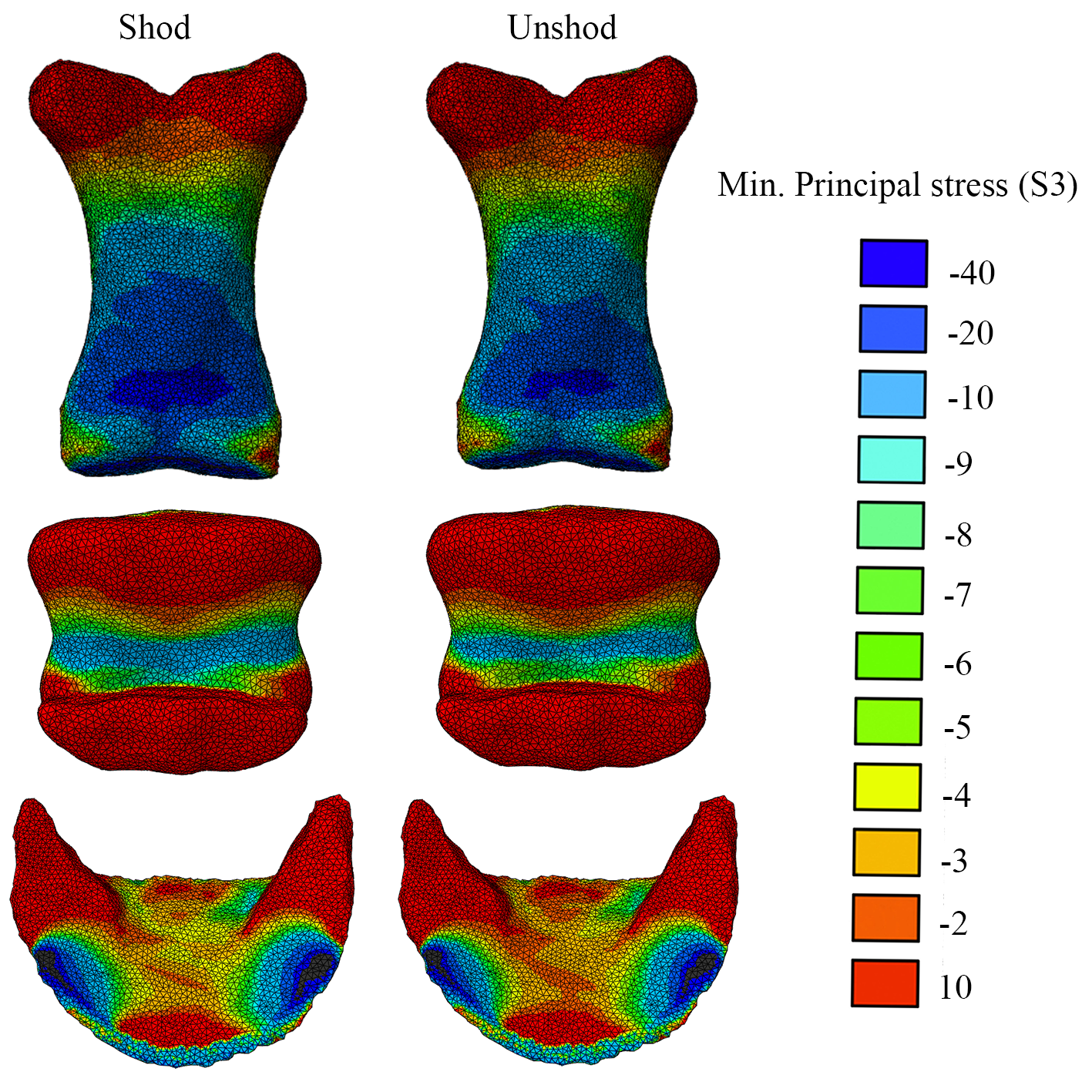

Supplement: Figure S6 — Bones shown from top to bottom are the P1, P2 and P3. Warm (red) and cold (blue) colours show higher and lower minimum principal stresses respectively. [file peerj-04-2164-s006.png]

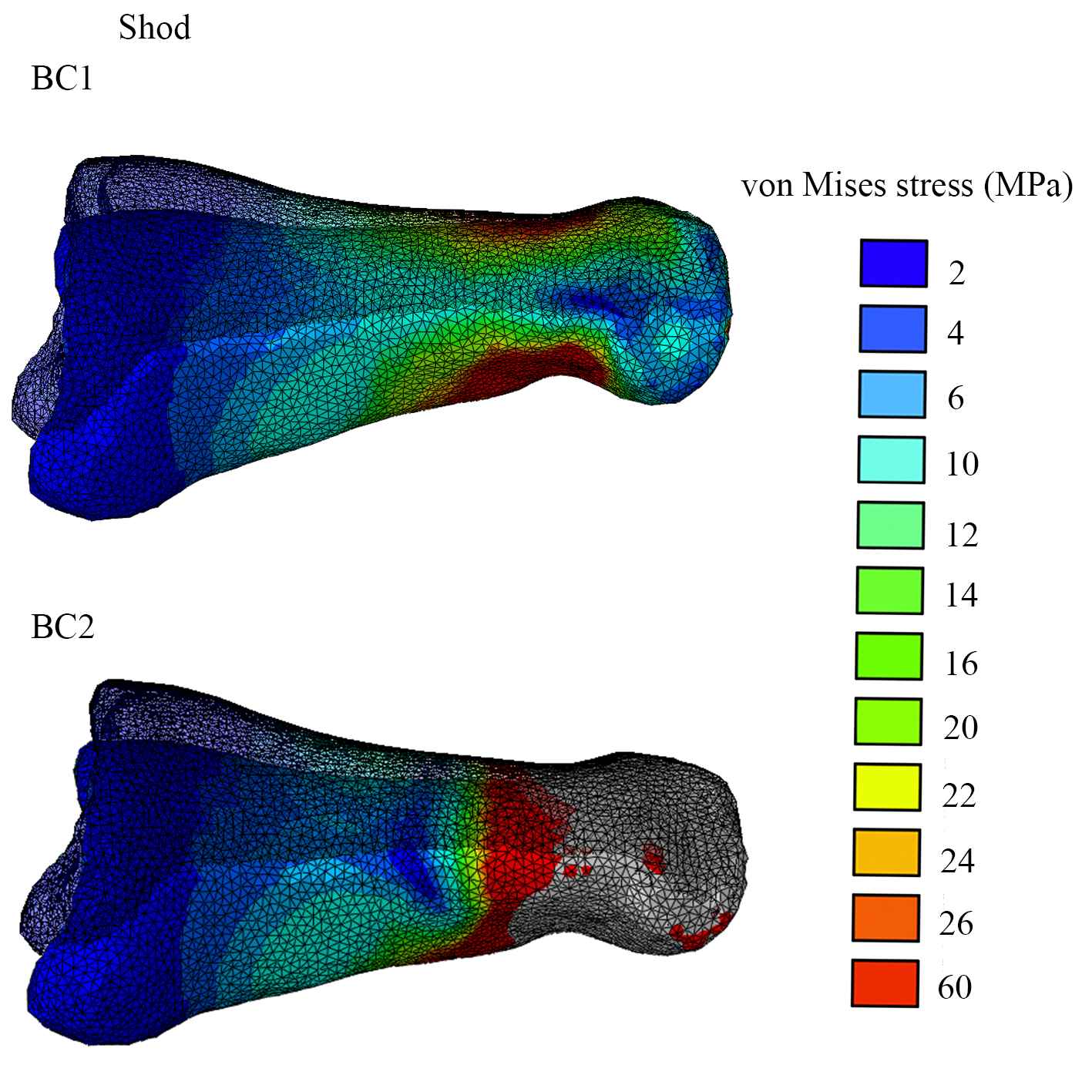

Supplement: Figure S7 — BC1 constraints are applied on the entire distal joint surface of the P1 and fixed in all axes. BC2 constraints involve fix constraining 3 nodes at the distal joint surface on the centroid axis of the load. The remainder nodes are fixed on the long axis of the bone only. Transparent and non-transparent images represent the undeformed and deformed bone respectively. Warm (red) and cold (blue) colours show higher and lower von Mises stresses respectively. Grey colours show von Mises stresses with magnitudes larger than 60 MPa. [file peerj-04-2164-s007.png]

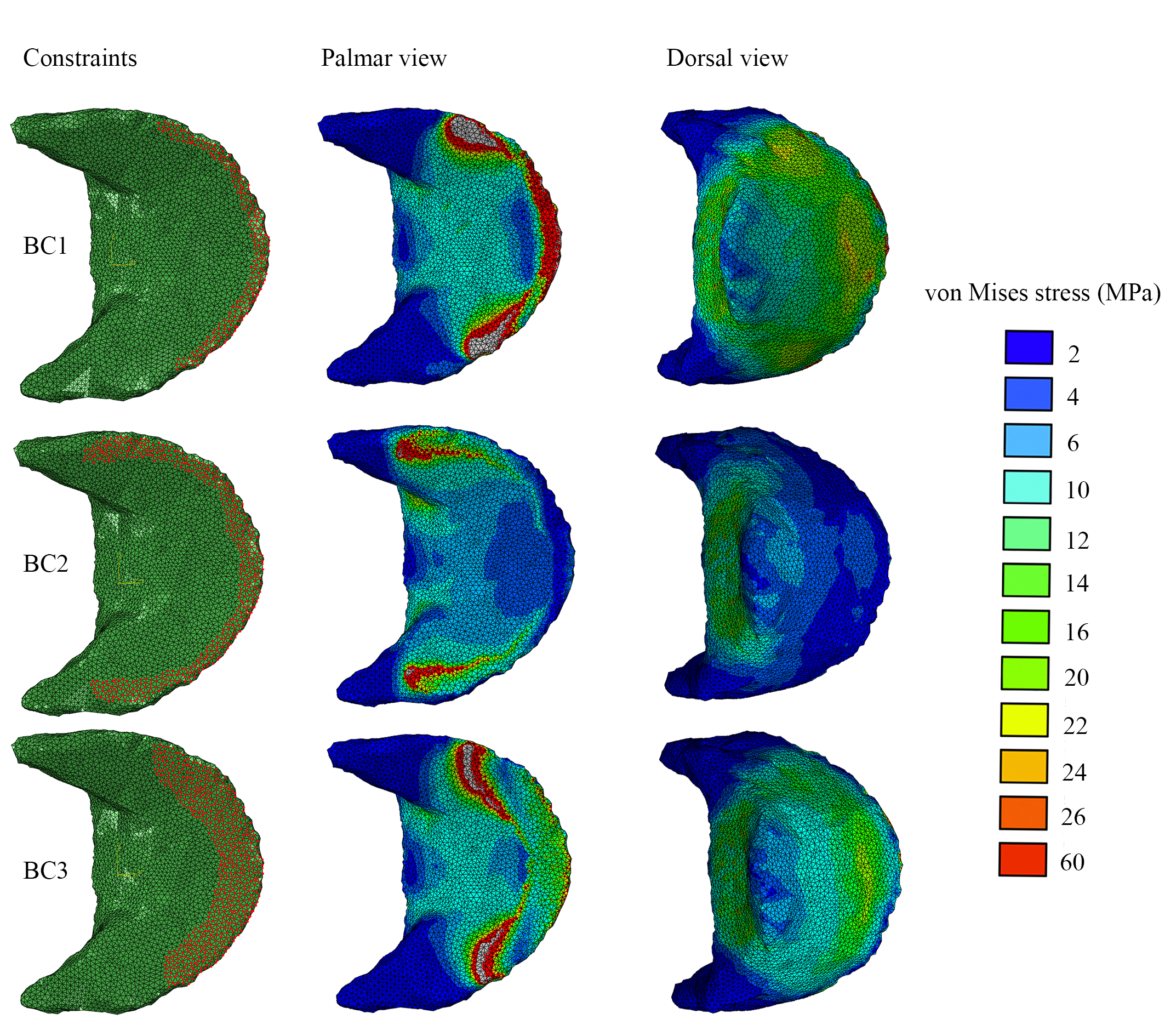

Supplement: Figure S8 — Warm (red) and cold (blue) colours show higher and lower von Mises stresses respectively. [file peerj-04-2164-s008.png]
